# QTL Mapping for Yield and Resistance against Mediterranean Corn Borer in Maize

**DOI:** 10.3389/fpls.2017.00698

**Published:** 2017-05-08

**Authors:** José C. Jiménez-Galindo, Bernardo Ordás, Ana Butrón, Luis F. Samayoa, Rosa A. Malvar

**Affiliations:** ^1^Misión Biológica de Galicia, Spanish National Research CouncilPontevedra, Spain; ^2^National Institute of Forestry, Agriculture and Livestock ResearchChihuahua, Mexico; ^3^Department of Crop Science, North Carolina State UniversityRaleigh, NC, USA

**Keywords:** insect resistance, quantitative trait loci, *Sesamia nonagrioides*, yield, *Zea mays*

## Abstract

**Introduction:** The Mediterranean corn borer (MCB), *Sesamia nonagrioides*, is a major pest of maize, *Zea mays*, in Mediterranean countries, inflicting significant kernel yield losses. For that reason, it necessary to know the genetic mechanisms that regulate the agronomic and resistance traits. A quantitative trait loci (QTL) mapping study for yield, resistance against MCB attack, and other relevant agronomic traits was performed using a recombinant inbred line (RIL) population derived from the cross A637 × A509 that is expected to segregate for yield, and ear, and stalk resistance to MCB. 171 RILs were evaluated in 2014 and 2015 at Pontevedra, Spain, along with the two parental inbreds A637 and A509 using a 13 × 14 single lattice design with two replications. A genetic map with 285 SNP markers was used for QTL analysis. Our objectives were to detect QTL for resistance to MCB and tolerance-related agronomic traits, to gain insights on the genetic relationship between resistance to MCB attack and yield, and to establish the best way for simultaneously improving yield and resistance to MCB.

**Results:** Twelve significant QTL were detected for agronomic and resistance traits. QTL at bins 1.10 and 5.04 were especially interesting because the same allelic variant at these QTL simultaneously improved yield and insect resistance. In contrast, in the region 8.04–8.05, QTL showed opposite effects for yield and resistance. Several QTL for indexes which combine yield and resistance traits were found especially in the region 10.02–10.03.

**Conclusions:** Selecting genotypes with the favorable allele of QTL on chromosome 5 (bin 5.01) will decrease tunnel length without affect yield, silking and plant height and QTL on the region 5.04 could be used to improve stalk resistance and yield simultaneously. An allele of QTL on bin 9.07 will increase ear resistance to MCB attack but it could produce later varieties while favorable allele in region 1.10 could improve ear and stalk resistance and yield without secondary negative effects. The region 8.03–8.05 mainly but also the region 10.02–10.03 and 5.04 may play an important role to elucidate the association between yield, other agronomic traits and MCB resistance.

## Introduction

The area planted with maize globally exceeds 179.9 million hectares, with a total annual production of 1013.62 million tons in 2014–2015. In the European Union, the area exceeds 9.57 million hectares, with a total production of 75.84 million tons in 2014–2015, COTRISA (2016). The corn borer belongs to various species of Lepidoptera, the most important of which are grouped into two families worldwide: Crambidae and Noctuidae. The main species within the Crambidae family are the European Corn Borer (ECB) *Ostrinia nubilalis* Hübner in North America, Europe, and North Africa; *Ostrinia furnacalis* Moth in Asia; *Diatraea saccharalis* Fabricius from the United States to Argentina; *Chilo partellus* Swinhoe in the southern United States, Central America, and the Caribbean; *Diatraea lineolata* Walker in Central America, the Caribbean, and South America; and *Diatraea grandiosella* Dyar in North and Central America (Ortega, 1987). The main species of the Noctuidae family include the Mediterranean corn borer (MCB) *Sesamia nonagrioides* Lefebvre in the Mediterranean region, *Busseola fusca* Fuller in Sub-Saharan Africa, and *Sesamia calamistis* Hampson in West Africa (Ortega, 1987). Some studies have reported losses caused by the corn borer of around 30% (Meissle et al., 2010). ECB, and MCB, are the most economically important maize pests in temperate areas of the northern hemisphere (Velasco et al., 2007). In Spain, MCB is the most damaging maize pest (Lopez et al., 2001). For example in northwestern Spain, the average yield loss for a set of 45 hybrids was 15% (Butrón et al., 1999).

The corn borer mainly feeds on the maize stem pith, resulting in lodging increase and in kernel yield losses because pith damage reduces the assimilate movement to developing cobs. Corn borers can also attack the ears, causing secondary fungal infections, which lead to contamination of the grain with mycotoxins that may affect human and animal health (Avantaggiato et al., 2003; Butrón et al., 2006).

Controlling these species with insecticides is difficult because eggs are protected by the leaf sheath and larvae have an endophytic behavior since the larvae tunnel throughout the stem from the first instar (Gonzalez-Nunez et al., 2000; Gonzalez-Cabrera et al., 2006). The use of transgenic maize (*Bt*-corn), which produces *Bt* toxins, is a good method for controlling the MCB, but transgenic crops are not authorized in most European countries and are banned in organic agriculture (Meissle et al., 2011). Since its registration in 1996, the use of *Bt*-corn has spread quickly. In 2014, 181.5 million hectares of genetically modified (GM) crops were planted globally (James, 2014). An estimated 0.1 million hectares of *Bt*-corn were grown in Spain in 2014 (James, 2014). However, recent studies have reported a reduced efficacy of *Bt* transgenes as some important pests have developed resistance to them (Campagne et al., 2013; Gonzalez-Cabrera et al., 2013). Insect resistance is the main risk for the success of this control tool (McGaughey and Whalon, 1992).

Exploitation of the genetic variability of maize for resistance to corn borer attack could be a complement and/or alternative to the use of *Bt-*corn. Indeed, some breeding programs developed in the Misión Biológica de Galicia (Pontevedra, Spain) have been successful in developing materials with partial resistance to the MCB (Sandoya et al., 2008; Butrón et al., 2014), although an indirect, undesirable effect was also achieved: yield reduction (Sandoya et al., 2008; Butrón et al., 2012, 2014).

Mediterranean corn borer (MCB) resistance, measured as shorter stem tunnels lengths made by MCB larvae, and yield have complex genetic architectures with many minor genes contributing to each trait (Cartea et al., 1999, 2001; Butrón et al., 2009, 2012). Therefore, searching QTL for resistance and yield in segregating populations for both traits would be a suitable method to discover genetic factors contributing to the negative relationship between resistance and yield. Further, the molecular markers derived from this kind of study could be used in a marker-assisted selection to simultaneously increase yield and resistance to the MCB. In previous mapping studies using bi-parental populations, no genetic correlation between yield and resistance to stem tunneling by MCB was reported and few QTL with opposite effects on yield and resistance were detected (Ordás et al., 2010; Samayoa et al., 2014, 2015b; Santiago et al., 2016). In contrast, the unfavorable genetic correlation between yield and resistance has been confirmed in the synthetic population EPS12 (Butrón et al., 2012). Differences between both types of studies could rely on the amount of available genetic variability for each trait. Genetic variability for yield and resistance in EPS12 was high because selection programs for improving each trait were successful (Butrón et al., 2012), but bi-parental mapping populations were designed to segregate for either yield or resistance but not for both simultaneously. (EP42 × EP39) F_2_ is a cross between Spanish susceptible × Spanish resistant lines and both have high yield losses. In other study, we used (A637 × EP42)F_2_ population, A637 is an American tolerant line while EP42 is a Spanish sensible line, both are susceptible to corn borer attack. In this study were used two American lines: A637 is tolerant to yield losses and stalk susceptible to corn borer attack while A509 has high yield losses but is resistant to larvae attack. Therefore, the bi-parental population used in the current study offers an additional advantage over the other previously used for revealing the genetic factors behind this unfavorable genetic correlation. The present population is derived from a cross between two lines with contrasting values for yield and resistance to stem tunneling by MCB (Butrón et al., 1998). In addition, the current mapping population is expected to segregate for ear resistance to MCB. To study ear and stalk resistance separately is important because heritability of ear resistance is complex and is not fully independent of stalk resistance (Cartea et al., 2001; Velasco et al., 2002, 2004).

The search of QTL for resistance and yield has focused more on individual characteristics and less on indexes that combined several traits. The use of indexes is common in breeding programs to improve two or more traits simultaneously (Sandoya et al., 2008). In this study, we looked for QTL that can be used to jointly improve resistance and yield.

To conclude, this study was undertaken to find QTL conferring resistance to stalk and ear damage made by MCB and QTL affecting agronomic traits (yield, silking, and plant height), to explore the genetic relationship between yield and resistance in a bi-parental population derived from the Corn Belt Dent heterotic group, and to establish the best way for simultaneously improving yield and resistance to MCB.

## Materials and methods

171 RILs (F_6_) derived from the cross A637 × A509 were used in this study. The inbred line A509 was sensitive (higher yield loss than the average) and resistant (shorter stem tunnel length and higher ear resistance than the average) to MCB attack, while the A637 line was tolerant (lower yield loss than the average) but susceptible (larger stem tunnel lengths and higher ear resistance than the average) to the MCB (Butrón et al., 1998). Parents and 171 RILs were genotyped using genotyping by sequencing (GBS) resulting in a total of 955,690 single nucleotide polymorphisms (SNPs), performed in the Institute of Biotechnology from the Cornell University. The version: Maize B73 RefGen_v2 were used for located the positions (Sen et al., 2010). The SNPs that were polymorphic between A637 and A509 and were sequenced in at least the 95% of the recombinant inbred lines (RIL) were then selected. SNP sites very close to each other do not provide additional useful information so we thinned out SNP sites based on a minimum distance of 1.5 Mb between adjacent sites to obtain a subset of 1,165 SNPs. Finally, RILs with a percentage of heterozygous markers ≥20% were removed, leaving 162 lines. Based on the conclusion of previous research comparing the use of high vs. low density marker map (Li et al., 2010; Stange et al., 2013) and our own experience we constructed a genetic map using MAPMAKER software (Lander et al., 2009) with a subset of 285 markers (see Availability of supporting data section), the resulting map had an average marker interval of 8.6 cM.

In 2014 and 2015, 171 RILs were evaluated along with the two parental inbreds A637 and A509 and nine inbreds as checks (EP125, EP39, EP42, EP47, EP53, EP86, F473, PB130, and W182B) using a 13 × 14 single lattice design with two replications. Trials were grown at Pontevedra, a location in northwestern Spain (42°25′ N, 8°38′ W, and 20 m above sea level). They were hand planted, and each experimental plot consisted of one row of 2.16 m length and 0.80 m between rows, with 13 two-kernel hills spaced 0.18 m apart. Plots were overplanted and thinned, obtaining a final density of ~70,000 plant ha^−1^. The evaluations were performed under artificial infestation with MCB eggs obtained at the Misión Biológica de Galicia by rearing the insects as described by Eizaguirre and Albajes (1992), and Khan and Saxena (1997). Before flowering, five normal and competitive plants per plot were infested with ~40 MCB eggs placed between the stem and the sheath of a basal leaf. Data collected were as follows: days to silking, measured as the days from planting to when 50% of plants within the plot showed silks; plant height, measured as the average length in centimeters from the ground to the top of five representative plants; kernel resistance to MCB larvae, measured by assessing the level of ear damage of the five infested plants using a subjective visual scale from 1 to 9 in which 1 indicated complete damaged and 9 indicated no damage (Malvar et al., 2004); tunnel length, stalks of 5 infested plants were longitudinally split to measure the length in centimeters of stem tunnels made by corn borers; larval stalk damage, calculated as tunnel length divided by plant height and multiplied by 100; and grain yield, estimated on a plot basis as Mg ha^−1^ at 140 g H_2_O kg^−1^.

The data from the experimental plots of the RILs were checked for normality using PROC UNIVARIATE of SAS software (SAS Institute, 2016). The mixed model procedure (PROC MIXED) of SAS were used for obtaining a best linear unbiased predictor (BLUP) of each line mean phenotypic value for individual and combined trial data. All factors: replications, blocks within replications, and RILs were considered as random effects. Heritabilities (ĥ^2^) across environments were estimated for each trait on a family-mean basis as described by Holland et al. (2002). The genotypic (*r*_*g*_) and phenotypic (*r*_*p*_) correlations between traits were computed following Holland (2006). All previous analyses were made in SAS software version 9.4.

QTL analysis was performed using the software package PlabMQTL (Utz, 2012). The composite interval mapping approach was conducted to detect QTL and to estimate QTL effects. According to a previously executed permutation test with 1,000 random reshuffles (Churchill and Doerge, 1994), a logarithm of odds (LOD) threshold of 3.55 with an empirical critical value of 30% was chosen to declare significant a putative QTL. The QTL mapping was conducted in two steps. In the first step, the entire genome was scanned to draw the LOD curves and identify the peaks where the putative QTL were located; in this preliminary fit, the additive effects of all preselected cofactors were estimated. In the second step, the QTL with the most important QTL detected in the previous step were screened using the Bayesian information criterion (BIC) as selection criteria for the stepwise regression procedure (Utz, 2012). Following (Utz et al., 2000), a 5-fold cross validation approach (CV/G) was employed for obtaining the unbiased predictor of the additive effect ($\hat{\alpha }$). For each trait, CV/G was performed with the whole data set (DS) of entry BLUPs across environments. A total of 130 entries were used as the estimation set (ES) for calibration, and 32 entries were used as the test set (TS) for validation. One thousand CV/G runs were performed to determine the QTL frequency and shrinkage of estimations for QTL effects detected in the original data set (Melchinger et al., 2004). The biases for the estimates of additive effects $\hat{\alpha }$_*i*_ were obtained with the formula: bias = (EffecTS-EfectES)/EffectTS.

The data set for each trait was standardized using the formula Z = $\frac{x-\mu }{\sigma }$, where *x* is the observed data, μ is the weighted mean of the trait, and σ is the standard deviation. From the standardized data, four indexes were calculated using standardized data: (1) Y+kr−tl (yield plus kernel resistance minus tunnel length); (2) Y−tl (yield minus tunnel length); (3) Y+kr (yield plus kernel resistance); and (4) Kr−tl (kernel resistance minus tunnel length) and QTL analysis for these indexes were carried out. Two or three traits were combined with the same weight to value together (1) yield and both resistances, (2) yield and stalk resistance, (3) yield and ear resistance, (4) stalk and ear resistance.

A QTL joint analysis to co-localizing QTL with additive effects on agronomic and resistance related traits was made using JointQTL option from PlabMQTL (Utz, 2012). A LOD threshold of 5.8. was used. The experiment-wise error expected was <0.10 on the basis of the meta-analyses performed with the LOD file generated by permutation analyses of single traits.

## Results

### Means, heritabilities, and correlations

Significant differences between A637 and A509 were found only for days to silking, and plant height (Table 1). Heritabilities ranged from low for kernel resistance (0.36) to high for plant height (0.88) (Table 1). Plant height showed moderate genetic correlation with yield (*r*_*g*_ = 0.65). The genetic correlation coefficients between tunnel length and yield (*r*_*g*_ = 0.59) and between tunnel length and plant height (*r*_*g*_ = 0.63) were moderate. The highest genetic correlation coefficient was between kernel resistance and silking (*r*_*g*_ = 0.86). On the other hand, phenotypic correlations were low; except the correlation between plant height and yield that exceeded 0.50 (Table 2).

**Table 1:** **Means and their standard errors (±SE), rank, and heritabilities (*h*^2^) of the RIL population derived from A637 × A509 for resistant and agronomic traits evaluated in a 2 years experiment under MCB infestation**.

	**Agronomic traits**			**Resistance traits**		
	**Silking (days)**	**Yield (Mg ha^−1^)**	**Plant height (cm)**	**Tunnel length (cm)**	**Stalk damage (%)**	**Kernel resistance (1–9)^a^**
**RILs**						
Mean	75.7	5.07	136.1	27.8	20.5	7.5
±SE	0.35	0.09	0.79	0.43	0.30	0.05
Rank	60–93	0.2–12.5	66–194	2.2–67.8	1.91–43.2	2.3–9.0
*h^2^*	0.88^b^	0.69^b^	0.83^b^	0.52^b^	0.43^b^	0.36^b^
**PARENTS**						
A509	73.7^b^	3.1^a^	119.5^b^	26.2^a^	22.1^a^	6.8^a^
A637	77.5^a^	4.1^a^	134.5^a^	38.1^a^	30.2^a^	7.0^a^
LSD	3.2	2.4	11.5	21.8	40.2	5.8
±SE	3.1	0.35	6.8	3.8	2.8	0.57

*Mean, LSD, and ±SE comparisons of the parental inbreeds are also shown. Heritabilities (h^2^) for each trait were estimated following Holland et al. (2002)*.

a *Kernel resistance was scored on a subjective visual scale from 1 to 9 in which 1 indicated complete damage and 9 indicated no damaged by the larvae*.

b *Significant difference from zero at 0.05 probability level*.

**Table 2:** **Genotypic (below) and phenotypic (above) correlation coefficients between agronomic and resistance to MCB traits recorded from RIL population derived from A637 × A509 evaluated in a 2 years experiment under MCB infestation**.

	**Silking**	**Yield**	**Plant height**	**Tunnel length**	**Stalk damage**	**Kernel resistance**
Silking		0.11	0.26	0.19	0.08	0.29
Yield	0.37		0.55	0.22	−0.004	0.31
Plant height	0.45	0.65		0.35	−0.08	0.30
Tunnel length	0.45	0.59	0.63		0.88	−0.02
Stalk damage	0.17	0.14	−0.13	0.66		−0.16
Kernel resistance	0.86	0.32	0.42	0.35	−0.02	

### QTL analysis: individual traits

**Figure 1:**
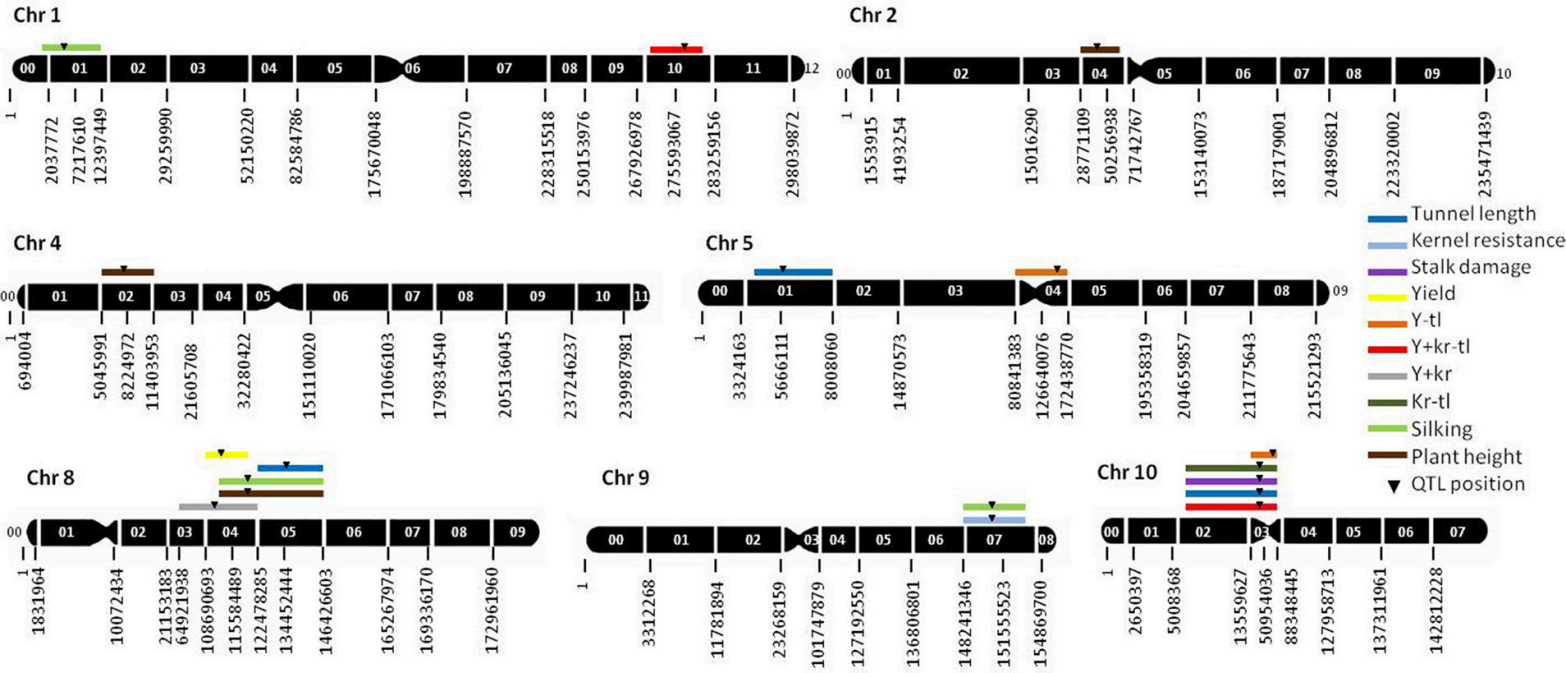
**Molecular linkeage map of maize based on 285 SNP markers and QTL positions**. The genetic map was constructed on 162 RILs derived from the A637 × A509. Only those chromosomes where significant QTL were located are shown. The black numbers below each chromosome indicate the position in bp of each SNP marker, and the white numbers on each chromosome indicate the bin number. 95% confidence intervals are indicated by the length of the QTL bar. Y−tl, yield minus tunnel length index; Y+kr−tl, yield plus kernel resistance minus tunnel length index; Kr−tl, kernel resistance minus tunnel length index; Y+kr, yield plus kernel resistance index. Stalk damage was calculated in percent respect to plant height.

The genetic map covered a length of 2372.1 cM. No segregation distortion from the expected ratio was observed for any marker. Twelve QTL were identified for individual traits related to resistance and agronomic traits (Table 3, Figure 1). Five QTL for resistance traits were found in this RIL population (Table 3, Figure 1). Three QTL for tunnel length were located on chromosomes 5 (bin 5.01), 8 (8.05), and 10 (10.02–10.03) and accounted for more than 44% of the total genotypic and 23% of the phenotypic variances. The absolute values of additive effects ranged from 0.79 to 1.12 cm for each QTL. One QTL for stalk damage was located on chromosome 10 (bin 10.02–10.03) and explained more than 30.3% of the genotypic and 13.1% of the phenotypic variance. The absolute value of additive effect for this QTL was 0.65%. One QTL for kernel resistance was located on chromosome 9 (bin 9.07), and explained the 20.1% of the genotypic and 7.2% of the phenotypic variances. The absolute value of additive effect for this QTL was 0.07 (scale from 1 to 9).

**Table 3:** **Summary of QTL mapped in the RIL population derived from A637 × A509 evaluated in a 2 year experiment under MCB infestation using a genetic map with an average interval between markers of 8.6 cM**.

**QTL position**					**Additive mean effect^e^**						
**Bin^a^**	**cM**	**95 %Cl^b^ (cM)**	**LOD^c^**	**Flanking markers' positions (bp)**	**DS^d^**	**ES**	**TS**	**Bias**	**Freq^f^**	**Phenot. V. (R^2^)^g^**	**Genetic V**.
**INDIVIDUAL TRAITS**											
**Tunnel length (cm)**											
5.01	31	24–40	3.94	4582822–6113453	−0.79	−0.90	−0.33	0.631	0.292	5.57	10.71
8.05	103	93–108	3.92	132298557–141751336	0.89	0.952	0.478	0.498	0.375	7.12	13.68
10.02–10.03	60	54–68	6.20	12084489–78061189	1.12	1.102	0.952	0.136	0.728	10.40	19.98
**Kernel Resistance^h^**											
9.07	141	136–145	5.44	150244089–151819320	0.067	0.069	0.047	0.319	0.498	7.20	20.13
**Stalk Damage (%)**											
10.02–10.03	60	54–67	5.50	12084489–78061189	0.652	0.657	0.603	0.082	0.874	13.10	30.37
**Yield (Mg ha^−1^)**											
8.04	91	88–97	8.23	113297722–119720129	0.488	0.507	0.468	0.077	0.790	15.90	23.01
**Silking (days)**											
1.01	14	9–19	8.42	4772203–6533500	−0.71	−0.74	−0.65	0.127	0.677	8.27	9.40
8.04–8.05	98	95–105	9.38	119720129–132298557	1.379	1.352	1.269	0.061	0.843	23.90	27.14
9.07	144	140–149	8.22	150244089–151819320	0.834	0.853	0.844	0.011	0.909	12.33	14.00
**Plant Height (cm)**											
2.04	113	109–125	4.11	40439132–43556793	4.028	4.164	1.652	0.603	0.323	5.08	7.94
4.02	65	56–72	4.30	8197994–11255195	4.885	4.941	3.286	0.335	0.342	6.90	10.79
8.04–8.05	99	94–105	8.27	119720129–132298557	5.478	5.445	4.801	0.118	0.674	9.12	14.27
**INDEXES**											
**Y+kr−tl**											
1.10	277	273–281	3.55	275382135–280088982	0.457	0.523	0.239	0.543	0.208	6.59	18.89
10.02–10.03	60	55–67	8.08	12084489–78061189	−0.61	−0.63	−0.59	0.063	0.854	10.91	18.28
**Y−tl**											
5.04	137	131–142	4.21	161477213–170970936	−0.30	−0.34	−0.14	0.587	0.282	5.64	11.78
10.03	65	52–70	4.30	78061189–86414417	−0.41	−0.43	−0.34	0.212	0.555	9.46	19.76
**Y+kr**											
8.03–8.04	85	80–89	3.63	94799312–113297722	0.314	0.355	0.152	0.572	0.234	5.00	8.87
**Kr−tl**											
10.02–10.03	61	56–67	7.60	12084489–78061189	−0.58	−0.58	−0.56	0.034	0.860	15.70	47.68

a *Bin locations were designed by an X.Y code, where X was the linkage group containing the bin, and Y was the location of the bin within the linkage group (Gardiner et al., 1993). Bins were based on the physical position of flanking markers. B73 RefGen_v3 was used for chromosome 1, and for chromosomes 2, 4, 5, 8, 9, and 10 B73 RefGen_v2 was used (available at: http://www.maizegdb.org/bin_viewer) (Sen et al., 2010)*.

b *95% confidence interval as explained in Utz (2012)*.

c *The LOD score in the LOD-profile used in scanning for QTL*.

d DS was the estimation for the complete data set; ES was the average value for the 1,000 estimation sets (80% of the genotypes of DS) in cross-validation; TS was the average value for the 1,000 validation sets (20% of the genotypes of DS) in cross validation; and bias was the estimation bias calculated as the difference between ES and TS estimations divided by the ES estimation

e *Additive effect of the QTL estimated as half the difference between the genotypic values of the two homozygotes. A positive estimation means that A637 carried the allele with higher value*.

f *Detection frequency of the QTL in the cross-validation test*.

g *Proportion of phenotypic variance explained by each QTL*.

h *Subjective visual resistance scale from 1 to 9 in which 1 indicated complete damage and 9 no damage by the larvae*.

*Kr−tl, kernel resistance minus tunnel length index; Y+kr, yield plus kernel resistance index; Y−tl, yield minus tunnel length index; Y+kr−tl, yield plus kernel resistance minus tunnel length index. Stalk damage was calculated as the percent of the plant height with damage*.

For yield, only one QTL located on chromosome 8 (bin 8.04) was detected. The proportion of genotypic and phenotypic variance explained by the QTL was 23.01 and 15.90%, respectively. The absolute value of additive effect was 0.49 Mg ha^−1^, and the detection frequency through the CV/G runs was 79%. Three QTL for silking were located on chromosomes 1 (bin 1.01), 8 (8.04–8.05), and 9 (9.07). These QTL accounted for 50.54% of the total genotypic and 44.50% of the total phenotypic variance. The absolute values of additive effects were 0.71, 1.38, and 0.83 days for the QTL on chromosomes 1 (1.01), 8 (8.04–8.05), and 9 (9.07), respectively. The percentage of bias estimation for additive effects was between 1.1 and 12.7%. The detection frequency in the cross validation of the QTL on chromosome 9 (9.07) was higher (0.90) than that observed for QTL on chromosomes 8 (8.04–8.05) and 1 (1.01). Three QTL for plant height were located on chromosomes 2 (bin 2.04), 4 (4.02), and 8 (8.04–8.05). These QTL accounted for 33.0% of the total genotypic and 21.1% of the total phenotypic variance. The absolute values of additive effects estimated were 4.03, 4.89, and 5.48 cm for the QTL on chromosomes 2 (bin 2.04), 4 (4.02), and 8 (8.04–8.05), respectively. The detection frequency in the cross validation of the QTL on chromosome 8 (8.04–8.05) was higher (0.67) than that observed for QTL on chromosomes 2 (bin 2.04) (0.32) and 4 (0.34).

### QTL analysis: indexes

Two QTL for the Y+kr−tl index were located on chromosomes 1 (bin 1.10) and 10 (10.02–10.03), and explained more than 37% of the genotypic and 17% of the phenotypic variance. The absolute values for additive effects for the QTL were 0.46 and 0.61 with bias estimations of 54 and 63%. Two QTL for the Y−tl index were located on chromosomes 5 (bin 5.04) and 10 (10.03) and explained more than 31% of the genotypic and 15% of the phenotypic variance. The absolute values of additive effects for the QTL in chromosomes 5 (bin 5.04) and 10 (10.03) were 0.30 and 0.41 with estimation biases of 21.2 and 58.7%, respectively. A QTL for Y+kr index was located in chromosome 8 (bin 8.03–8.04) and accounted for 8.87% of the genotypic and 5.0% of the phenotypic variances, respectively. The absolute value of the additive effect estimated TS for this QTL was 0.31. Another QTL located on chromosome 10 (bin 10.02–10.03) for Kr-tl index accounted for 47.6% of the genotypic and 15.7% of the phenotypic variances. The absolute value of the additive effect estimated for this QTL was 0.58.

### Co-localization of QTL for agronomic and resistance-related traits: joint analysis

Analyzing the joint effect of two traits confirmed that the QTL on bins 8.04–8.05 significantly increased yield and tunnel length (Table 4). The joint analysis also showed co-localization of QTL at bins 8.04–8.05 for significantly increased silking and yield, yield and plant height, silking and tunnel length, plant height and tunnel length, yield and kernel resistance, and tunnel length and kernel resistance. On chromosome 9, the QTL at bin 9.07 significantly increased silking and kernel resistance. And finally on chromosome 10, the QTL at bin 10.02–10.03 significantly increased tunnel length and kernel resistance (Table 4).

**Table 4:** **Joint list of QTL from the Join analysis using LOD score >5.8 (90% of confiability) mapped in the RIL population derived from A637 × A509 evaluated in a 2 year experiment under MCB infestation**.

**Chr**	**Position**	**Trait 1**	**Effect**	**Trait 2**	**Effect**
8	95	Yield (Mg ha^−1^)	0.498^**^	Tunnel length (cm)	0.849^**^
8	96	Silking (days)	1.368^**^	Yield (Mg ha^−1^)	0.522^**^
8	96	Yield (Mg ha^−1^)	0.540^**^	Plant height (cm)	5.993^**^
8	99	Silking (days)	1.370^**^	Tunnel length (cm)	0.948^**^
8	99	Plant height (cm)	5.784^**^	Tunnel length (cm)	0.920^**^
8	96	Yield (Mg ha^−1^)	0.492^**^	Kernel resistance (1–9)^a^	0.049^**^
8	103	Tunnel length (cm)	0.861^**^	Kernel resistance (1–9)^a^	0.058^**^
9	144	Silking (days)	0.806^**^	Kernel resistance (1–9)^a^	0.057^**^
10	59	Tunnel length (cm)	1.149^**^	Kernel resistance (1–9)^a^	−0.046^**^

a *Kernel resistance was scored on a subjective visual scale from 1 to 9 in which 1 indicated complete damage and 9 indicated no damaged by the larvae*.

** *Significantly different from zero at P < 0.01*.

## Discussion

In this study, an intermediate value of *h*^2^ = 0.52 was obtained for tunnel length, similar to the *h*^2^ = 0.49 obtained with other RIL populations infested with the MCB (Samayoa et al., 2015b). The heritability for kernel resistance obtained in this study (0.39), was also in the range of values obtained by other researchers (Ordás et al., 2009; Samayoa et al., 2014, 2015b; Santiago et al., 2016). Previous studies have shown that heritability of stalk resistance to MCB varies greatly depending on the growing environments and RIL populations, ranging from *h*^2^ = 0.12 (Santiago et al., 2016) to *h*^2^ = 0.77 (Ordás et al., 2009). Further, the heritabilities for agronomic traits (*h*^2^ = 0.69 to *h*^2^ = 0.88) were similar to those obtained by other authors using diverse RIL populations under infestation with corn borers (Bohn et al., 1996, 2000; Papst et al., 2001; Ordás et al., 2009; Samayoa et al., 2014, 2015b), except for Santiago et al. (2016), who found heritabilities from *h*^2^ = 0.12 to *h*^2^ = 0.70. Tunnel length had an intermediate heritability (*h*^2^ = 0.52), was lower than the heritabilities for agronomic traits but enough high to wait positive results in selection programs to reduce larvae damage. Tunnel length trait has associated a high experimental error because it is affected by plant genotype, larvae pressure and environment and it is more difficult to measure than some agronomic traits as plant height. So, resistance traits show generally low heritabilities.

The genetic correlation coefficients between tunnel length and agronomic traits in this study indicates that susceptible genotypes would have greater yield, taller plants, and a delayed maturity than those resistant to the MCB as it has been previously suggested by Butrón et al. (1999). This agrees with the results in other studies (Samayoa et al., 2014; Malvar et al., 1993). However, it should be noted that some authors (Ordás et al., 2010; Samayoa et al., 2015b) did not find an appreciable genetic correlation between tunnel length and yield. Ordás et al. (2013) found a relationship between corn borer damage and the number of days from flowering to infestation. A later stage of plant development at the moment of corn borer infestation reduced larval damage. Early sowing was therefore recommended so that plant tissues would be mature at the moment of insect attack. In this study, the artificial infestation was carried out the same day in all lines. So, MCB larvae fed on immature attractive tissue for longer time in late lines than in early ones. Thus, longer tunnels could be produced by larvae in late varieties.

QTL of MCB resistance appeared in regions where none had been found previously, as is usual when we look for QTL for resistance in biparental populations, it is reinforcing the hypothesis of the polygenic character of this trait. However, the QTL for MCB tunnel length located at bins 8.04–8.05 was already reported in a previous study with RILs derived from a cross between Spanish inbred lines (Ordás et al., 2010). For the QTL at bins 8.05 and 10.02–10.03, alleles for reducing tunnel length came from the line A509. This line was classified as resistant by Butrón et al. (1998). However, the susceptible line A637 showed favorable allele of resistance for the QTL at bin 5.01. These results indicate that resistant genotypes could be improved by introducing complementary allelic variants that could come from other resistant genotypes but also from susceptible one. The additive effects for the three QTL detected for tunnel length (α = 0.79–1.12) were similar to most additive effects reported until now (α = 0.5–1.2), except those found in the RILs derived from B73 × CML103, which were substantially higher (α = 3.0–4.1). The differences in tunnel length between B73 and CML103 were much greater than those between any other pair of parental lines, which would explain these higher additive effects detected in the RILs released from these parents (Samayoa et al., 2015b). The most reliable QTL for tunnel length was at bins 10.02–10.03, where the CV/G analysis revealed that it was detected in 73% of CV/G runs. This QTL on chromosome 10 (10.02–10.03) was also found to be related to stalk damage.

For kernel resistance, a single QTL located on chromosome 9 at bin 9.07 was detected and explained more than 20% of the genetic variance. In the same chromosome, at bins 9.02–9.04, Santiago et al. (2016) already reported a QTL for kernel resistance that explained about 20% of the genetic variance. Other QTL studies have reported QTL for kernel resistance to the MCB on chromosomes 2, 3, 4, 5, 6, 7, and 8 (Ordás et al., 2009; Samayoa et al., 2014, 2015a; Santiago et al., 2016). The additive effect for kernel resistance in this study was 0.04, lower that those reported by other authors, where it ranged from 0.15 to 0.40 (Ordás et al., 2009; Samayoa et al., 2015a). The effect is small because there was low genetic variability between RIls although significant differences among them were found. A single QTL for yield was located at bin 8.04. This region of chromosome 8 (bin 8.04) could contain key genes for yield because QTL with additive effect on yield and/or with augmented dominance effect on yield heterosis have been located across different populations, this region could be considered as a hot spot (Ordás et al., 2010; Samayoa et al., 2017). A QTL for silking at bins 8.04–8.05 was found and proved very important because it was detected in 84% of the CV/G runs. This corresponds with results obtained by other authors who found remarkable QTL for silking at bin 8.05 in different backgrounds (Ordás et al., 2009, 2010; Samayoa et al., 2014). Probably refers to the QTL Vegetative to generative transition1 (*Vgt1*) locus on chromosome 8 at 132 Mb, a major QTL for flowering time, which has been cloned in maize (Salvi et al., 2002, 2007).

This study identified three QTL for plant height on chromosomes 2, 4, and 8 at bins 2.04, 4.02, and 8.04–8.05, respectively. Veldboom and Lee (1996) already found a QTL for plant height in the same region of chromosome 2; Cai et al. (2012a) a QTL at bin 4.01; Samayoa et al. (2014) at bin 8.05; and Wei et al. (2009) a QTL at bin 8.03. Chen et al. (2012) confirmed the position of gen *bm6* new *bm* mutation (Ali et al., 2010) for plant height and cell wall digestibility at bin 2.02.

Indexes have been widely used to improve two or more traits simultaneously. Sandoya et al. (2008) used an index to improve resistance while maintaining yield in EPS12 population. The method was effective in the first three cycles of selection but yield decreased in subsequent cycles (Butrón et al., 2012). Unfavorable genetic correlation between stalk tunnel length and yield was confirmed and those results advise against phenotypic selection for reduced tunnel length by MCB or any index involving yield and tunnel length. An alternative to phenotypic selection could be to use marker-assisted selection (MAS) for QTL that affect MCB resistance and yield. So, we have looked for QTL for different indexes combining stalk and ear resistance and yield.

QTL for indexes should be explored to improve several traits simultaneously. The final fit for the Y+kr−tl index revealed the presence of two QTL at bin 1.10, and 10.02–10.03. The QTL found for index allows identifying the favorable allele to improve both resistance and yield simultaneously. QTL for Individual traits, which are part of the index, had previously been found at bin 1.10 but only for one trait and in different RILs populations. Thus, Samayoa et al. (2015b) found a QTL related to grain yield at bin 1.10, Quero et al. (2004) found one QTL related to kernel resistance. In this population no significant QTL was found at bin 1.10 neither for the MCB resistances nor for the yield. On the other hand, Cai et al. (2012b) found a QTL for grain yield at bin 10.03–10.04 that could be related to the QTL for Y+kr−tl at bins 10.02–10.03. We also found a significant QTL for tunnel length at this region. Both QTL for index probably could be used in MAS to improve yield and stalk and ear resistance.

The final fit for the Y-tl index revealed two QTL on chromosomes 5 and 10 (bins 5.04 and 10.03, respectively). The favorable allele at bin 5.04 and at bin 10.03 came from A509 and they will be useful for increasing yield and reducing tunnel length in a MAS program, these QTL did not affect kernel resistance. Samayoa et al. (2014) found a QTL for yield under MCB infestation at bin 5.03 in a RIL population derived from EP42 × A637, with A637 providing the favorable allele. The bin 5.04 could harbor genetic variants controlling both yield and stalk resistance since several authors previously found QTL for these traits in the same region (bins 5.03–5.04) in different bi-parental populations (Cai et al., 2012a,b). Therefore, it could be interesting to conduct further studies in this region to elucidate the relationships between yield and stalk resistance. One QTL for the Y+kr index was found on chromosome 8 (bin 8.03–8.04) (85 cM), but it was near to QTL for tunnel length (bin 8.05). The favorable allele increased yield and kernel resistance, but increased tunnel length.

The joint analysis corroborated the results of the correlation analysis. Associations between yield and tunnel length were found, as well as QTL for silking and plant height. For all joint QTL, A637 allele produced later maturing and taller plants with greater yield at harvest and longer tunnels than those produced by A509 allele. Therefore, other characteristics, besides yield, such as plant height should be examined to clarify the negative relationship between yield and resistance. It must be emphasized that similar associations between resistance and agronomic traits have been found at bins 8.04–8.05 in lines with different genetic backgrounds, which makes that region especially interesting for further studies about relationship between MCB resistance and yield.

## Conclusions

Three QTL for tunnel length were found on chromosomes 5, 8, and 10 (at bins 5.01, 8.05, and 10.02–10.03, respectively), one for kernel resistance on chromosome 9 (bin 9.07), one for stalk damage on chromosome 10 (bin 10.02–10.03), one for yield on chromosome 8 (bin 8.04), three for silking on chromosomes 1, 8, and 9 (bins 1.01, 8.04–8.05, and 9.07, respectively), three for plant height on chromosomes 2, 4, and 8 (bin 2.04, 4.02, and 8.04–8.05, respectively). At the same time, two QTL for Y+kr−tl index were found on chromosomes 1 and 10 (bin 1.10, and 10.02–10.03), two QTL for Y−tl on chromosomes 5 and 10 (bin 5.04, and 10.03), one QTL for Y+kr on chromosome 8 (bin 8.03–8.04), and finally one QTL for Kr-tl on chromosome 10 (bin 10.02–10.03).

The region on chromosome 8 (bin 8.03–8.05) mainly but also the region on chromosome 10 (bin 10.02–10.03) and chromosome 5 (bin 5.04) may be the keys to elucidate the association between yield, other agronomic traits and MCB resistance. So, we recommend fine mapping in these regions for looking for resistance to corn borers and yield genes.

Selecting genotypes with the favorable allele of QTL on chromosome 5 (bin 5.01) decreased tunnel length. This same QTL did not affect yield, silking and plant height. The same way, the QTL on chromosome 9 (bin 9.07) could be used to improve ear resistance to MCB attack but it could produce later varieties.

The calculation of indexes from the original data set could help plant geneticists and plant breeders to identify QTL which could be used in MAS programs to improve yield and kernel resistance and decrease tunnel length. The region in chromosome 5 (bin 5.04) could be used to improve stalk resistance and yield while regions in chromosome 1 (bin 1.10) and 10 (bin 10.02–10.03) could improve also ear resistance.

## Author contributions

JCJG carried out field experiments, performed statistical analysis of the data, and drafted the initial manuscript. AB and RAM created and generated the vegetal materials. RAM and BO, advisors, assisted JCJG in the field experiment design, data collection, and statistical analysis. AB and LFS participated in the data collection and statistical analysis. All authors have read and approved the final version of the manuscript.

## Funding

This research was funded by the Plan Estatal de Ciencia y Tecnologia de España within the projects AGL2012-33415 and AGL2015-67313-C2-1-R, both of which were co-financed with European Union funds under the FEDER program, and the project: IN607A/013 funded by Autonomous Government of Galicia, Spain.

### Conflict of interest statement

The authors declare that the research was conducted in the absence of any commercial or financial relationships that could be construed as a potential conflict of interest.

## Abbreviations

BIC: Bayesian information criterion
BLUP: best linear unbiased predictor
CV/G: cross validation approach
DS: data set
ECB: European corn borer
ES: estimation set
MAS: Marker-assisted selection
MCB: Mediterranean corn borer
QTL: quantitative trait loci
RIL: recombinant inbreed lines
SNP: single nucleotide polymorphism
TS: test set
GM: genetically modified
CIM: composite interval mapping
GBS: genotyping by sequencing
SNPs: single nucleotide polymorphisms
LOD: logarithm of odds
cm: centimeters
cM: centimorgans.
